# Simultaneous assessment of iodine, iron, vitamin A, malarial antigenemia, and inflammation status biomarkers via a multiplex immunoassay method on a population of pregnant women from Niger

**DOI:** 10.1371/journal.pone.0185868

**Published:** 2017-10-05

**Authors:** Eleanor Brindle, Lorraine Lillis, Rebecca Barney, Sonja Y. Hess, K. Ryan Wessells, Césaire T. Ouédraogo, Sara Stinca, Michael Kalnoky, Roger Peck, Abby Tyler, Christopher Lyman, David S. Boyle

**Affiliations:** 1 Center for Studies in Demography and Ecology, University of Washington, Seattle, WA, United States of America; 2 PATH, Seattle, WA, United States of America; 3 Program in International and Community Nutrition, Department of Nutrition, University of California, Davis, CA, United States of America; 4 Helen Keller International, Niamey, Niger; 5 Laboratory of Human Nutrition, Swiss Federal Institute of Technology, Zurich, Switzerland; 6 Quansys Biosciences, Logan, Utah, United States of America; California State University Fresno, UNITED STATES

## Abstract

Deficiencies of vitamin A, iron, and iodine are major public health concerns in many low- and middle-income countries, but information on their status in populations is often lacking due to high costs and logistical challenges associated with assessing micronutrient status. Accurate, user-friendly, and low-cost analytical tools are needed to allow large-scale population surveys on micronutrient status. We present the expansion of a 7-plex protein microarray tool for the simultaneous measurement of up to seven biomarkers with relevance to the assessment of the key micronutrients iron, iodine, and vitamin A, and inflammation and malaria biomarkers: α-1-acid glycoprotein, C-reactive protein, ferritin, retinol binding protein 4, soluble transferrin receptor, thyroglobulin, and histidine-rich protein II. Assay performance was assessed using international reference standards and then verified by comparing the multiplexed and conventional immunoassay results on a training panel of plasma samples collected from US adults. These data were used to assign nominal concentrations to the calibrators of the assay to further improve performance which was then assessed by interrogating plasma samples from a cohort of pregnant women from Niger. The correlation between assays for each biomarker measured from this cohort was typically good, with the exception of thyroglobulin, and the sensitivity ranged from 74% to 93%, and specificity from 81% to 98%. The 7-Plex micronutrient assay has the potential for use as an affordable tool for population surveillance of vitamin A, iron, and iodine deficiencies as well as falciparum malarial parasitemia infectivity and inflammation. The assay is easy-to-use, requires minimal sample volume, and is scalable, rapid, and accurate—needing only a low-cost reader and basic equipment present in most reference laboratory settings and so may be employed by low and middle income countries for micronutrient surveillance to inform on status in key populations. Micronutrient deficiencies including iron, iodine, and vitamin A affect a significant portion of the world’s population. Efforts to assess the prevalence of these deficiencies in vulnerable populations are challenging, partly due to measurement tools that are inadequate for assessing multiple micronutrients in large-scale population surveys. We have developed a 7-plex immunoassay for the simultaneous measurement of seven biomarkers relevant to assessing iodine, iron, and vitamin A status, inflammation and *Plasmodium falciparum* parasitemia by measuring levels of thyroglobulin, ferritin, soluble transferrin receptor, retinol binding protein 4, α-1-acid glycoprotein, C-reactive protein, and histidine-rich protein II. This 7-plex immunoassay technique has potential as a rapid and effective tool for use in large-scale surveys and assessments of nutrition intervention programs in low- and middle-income countries.

## Introduction

Micronutrient (MN) deficiencies, particularly among young children and women of reproductive age, have long been recognized as a significant public health burden in low- and middle-income countries (LMICs); however, despite the implementation of health surveillance systems since the 1980’s, relatively limited literature has been published on micronutrient surveys in low-income countries [1–3]. MN surveillance is necessary to provide early warning of risk of deficiency, to develop appropriate policies, to guide the planning and implementation of nutritional and public health intervention programs, and to evaluate the impact of these interventions [4]. The accurate assessment of MN status to indicate prevalence and severity of deficiencies is presently challenged in part by the high costs and in part by logistical barriers associated with biological sample collection, and subsequent measurement of biomarkers of multiple micronutrients [4]. This study expands upon our previous work that described a multiplexed tool for the simultaneous assessment of vitamin A (VA), iron deficiency (ID), and acute phase proteins (APP) by improving assay components and adding further biochemical indicators of iodine deficiency and recent or current malarial infection with *Plasmodium falciparum* [5].

The two most commonly used biomarkers to assess iron status are ferritin and the soluble transferrin receptor (sTfR), which are typically measured in serum or plasma via enzyme-linked immunosorbent assay (ELISA)-based methods [6–8]. The serum concentration of ferritin—the major carrier protein of iron in blood—declines early in the development of iron deficiency [9–11] but also increases in response to inflammation independent of iron status, confounding proper assessment of ID in individuals experiencing inflammation [9]. Serum levels of sTfR increase in ID [12,13], confirmed by the evaluation of stainable marrow iron [14]; although, unlike ferritin, sTfR levels are not directly influenced by inflammation [15]. However, a recent review from the Biomarkers Reflecting Inflammation and Nutritional Determinants of Anemia (BRINDA) project noted that sTfR levels may be affected by inflammation via alternative mechanisms, such as iron-limited erythropoiesis and hypoxemia [16], and others have observed sTfR elevation with asymptomatic malaria [17]. The combined measurement of both ferritin and sTfR allows the data to be further displayed as the ratio of sTfR/ferritin [18,19], sTfR/log ferritin [20], or log (sTfR/ferritin) [18] to reflect depletion of body iron stores [12].

In addition to retinol, vitamin A deficiency (VAD) can be identified by assessing the levels of an accepted surrogate biomarker, retinol binding protein 4 (RBP), which is more stable than retinol and less technically challenging to measure [21]. Retinol and RBP decrease in VAD, but they are under homeostatic control and thus are not directly reflective of liver VA stores when replete [22,23]. However, they are indicative of low status when liver VA levels are very low. RBP is a negative acute phase inflammatory reactant where inflammation suppresses levels of RBP in the serum (plasma) [24].

Biomarkers of iodine status have focused on urinary iodine concentration and thyroid function, such as the presence of goiter or thyroid hormone measurements, including thyroid stimulating hormone, thyroxine, triiodothyronine, and thyroglobulin (Tg) [25]. Tg is a sensitive indicator of iodine status, reflecting both iodine deficiency and excess [26,27]. Quantitative measurement of Tg has been demonstrated in serum, plasma, and dried blood spots via immunoassays [27,28].Tg is unaffected by inflammation.

The suppression of RBP and increased serum ferritin concentration during inflammation are indicative of the sequestration of micronutrients during inflammation (including sub clinical inflammation) and malaria infection [29]. Thus, C-reactive protein (CRP) and α-1-acid glycoprotein (AGP) are commonly measured alongside MN biomarkers to adjust for the influence of inflammation. In malaria endemic regions, malaria infection often co-exists with MN deficiencies, particularly with ID, and so simultaneous assessment of malaria status (including asymptomatic malaria) with MN status is useful in settings where *P*. *falciparum* is prevalent [17]. *P*. *falciparum*, the most common and most virulent of the malarial parasites [30], can typically be detected through measurement of histidine-rich protein II (HRP2) and therefore serves as an indicator of current or recent infection [31]. HRP2 is a practical biochemical indicator for integration with MN surveillance, as it can also be measured via an immunoassay.

A recent study in Burkina Faso noted that asymptomatic malarial infections in high-prevalence areas had an additive effect to elevated APP, resulting in inaccurate estimates of MN deficiency prevalence when only APP adjustments were considered [17]. The Biomarkers Reflecting Inflammation and Nutritional Determinants of Anemia (BRINDA) project have released a series of reports describing the outputs of various analyses by which to adjust for total body iron, ferritin, RBP and sTfR levels when in conjunction with elevated APPs and malarial infection in preschool children and/or women of childbearing age [32–35].

For more effective population-based assessments of MN status, there is a need for tools that permit nutrition programs and researchers to collect, process, and accurately analyze MN status data in country, ideally for biomarkers of multiple micronutrients simultaneously. Thus, practical considerations, such as easy sample collection and processing, cold chain during transportation, and simplified testing in the laboratory are of heightened importance. Ideally, a method should not require multiple different individual assays to quantify MN biomarkers, permitting the use of a small sample volume to generate results and a more rapid processing time. We have developed a multiplexed immunoassay to quantify MN biomarkers using the Q-Plex™ platform (Quansys Biosciences, Logan, Utah, USA). This format significantly reduces the overall number of assays that need to be performed to provide the same or extra data but with less cost compared to commercial monoplexed immunoassays, labor, and time required to test large numbers of samples as compared to multiple analyses of individual biomarkers [5,36]. The multiplexed assay uses a protocol that is very similar to conventional ELISAs, a commonly used technique in most reference laboratories. The raw data are analyzed using software that permits multiple measurements and calculations to be automatically performed. The raw and processed data are stored in a single electronic file. Each file is identically formatted and so multiple datasets can be easily pooled into a master data file with less risk of data entry error.

In this study, we describe the development of a revised and expanded version of the original multiplexed MN assessment tool [5] to the Human Micronutrient Assay (7-Plex) that includes 1) the quantitative measurement of Tg and HRP2 biomarkers; 2) improvements to the performance of the original assay [5], including revision of the sTfR assay using different antibodies, a full revision of the calibration of each assay, new calibrator material, and adding a reference spot to assist with image capture prior to data analysis; and 3) the refinement of the new assay design using reference standards and a USA donor training panel screened with predicate tests for the biomarkers assayed by the 7-plex. The panel allowed for the iterative design changes to the 7-plex assay. The performance of the 7-Plex assay was then finally assessed using samples obtained from the NiMaNu cohort, comprised from a cohort of pregnant women from Niger [37]. The larger NiMaNu panel represents a key population that the 7-plex assay is intended to survey and had all of the 7-plex biomarkers measured by predicate assays. Therefore the NiMaNu panel presented an excellent opportunity by which to compare the performance of the 7-plex assay with other current tools used to measure micronutrient status and inflammation.

## Materials and methods

### Human micronutrient (7-Plex) assay development

Quansys Biosciences (Logan, UT, USA) conducted the prototype assay development using their proprietary Q-Plex™ technology described in detail elsewhere [5,38]. The original development work was expanded upon with the addition of quantitative assays HRP2 and Tg to the existing 5-plex array, accompanied by a redesign of the sTfR assay component as a key antibody is no longer commercially available [5]. Several candidate antibodies specific to HRP2, Tg, and sTfR were screened for initial performance on the Q-Plex™ technology to establish analytical-specificity and -sensitivity for each antibody [39]. Selection of optimal matched pairs of antibodies (typically monoclonal) was done by printing each candidate antibody at various concentrations on the bottom of flat-bottomed 96-well plates to assess their avidity and specificity as capture moieties. In addition, each candidate antibody was also biotin labeled (NHS-Biotin, Invitrogen, Carlsbad, CA, USA) to be screened as a detector in a sandwich immunoassay format. Matched optimal antibody pairs and assay conditions were identified using a checkerboard pattern to titrate antigen, detection antibody, and capture antibody concentrations. Antibody matched pairs were evaluated for sensitivity, specificity, precision, parallelism, and sample correlation as described in the Quansys development phases I and II [40]. Preliminary evaluation of the new assays for Tg and sTfR used commercially available ELISAs as reference assays (Tg [EIA-4126 from DRG International, Springfield, NJ, USA] and sTfR [DTFR1 from R&D Systems Inc., Minneapolis, MN, USA]). Following the optimization of the new assays for HRP2, Tg, and sTfR, the assays were integrated into a 7-Plex assay format with the 4 assays (RBP, CRP, AGP, and ferritin) developed previously [5].

**Fig 1:**
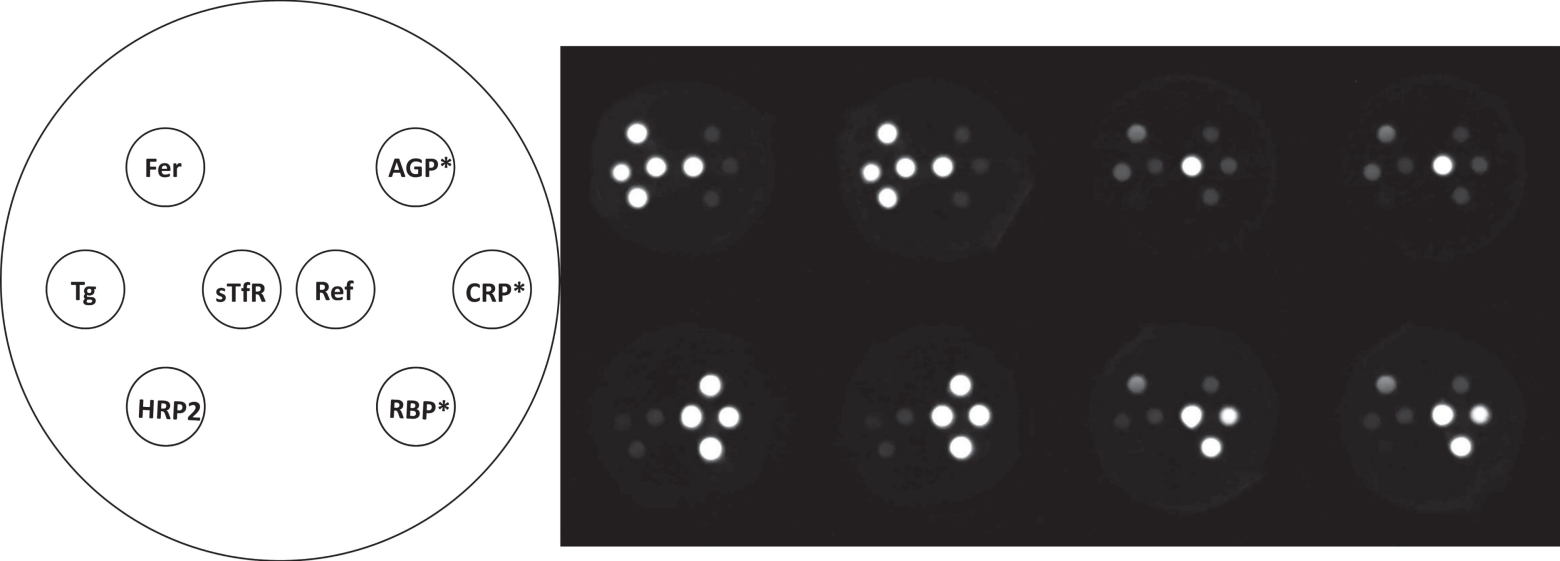
The orientation of assays and a reference spot on the 7-Plex planar array displayed in each well of a 96-well plate. The image on the right is an example of test wells and the levels of chemiluminescence of each test spot on the 7-Plex array when challenged with different levels of analyte: 8 wells with 4 duplicate samples screened using a high calibrator (top left) where the sandwich assay spots give a bright signal and the competitive assay spots are muted, negative controls (bottom left) where the competitive assays have a strong signal whilst the sandwich assays are faint, and two samples (top and bottom right). The reference spot in each well remains at a similar intensity across all wells shown. AGP, α-1-acid glycoprotein; CRP, C-reactive protein; Fer, ferritin; HRP2, histidine rich protein II; Ref, reference spot; RBP, retinol binding protein 4; sTfR, soluble transferrin receptor; Tg, thyroglobulin. ^a^ Indicates a competitive ELISA format, other assays are in a sandwich format.

In the new 7-Plex, the optimal capture antibodies for all seven analytes were printed in an array on the bottom of flat-bottomed 96-well plates. A reference spot producing a high intensity signal was also printed in each well to assist with alignment of the final plate image in the Q-View software prior to analysis (Fig 1). To enable the multiplexed detection of biomarkers in the range from ng/mL to mg/mL, the assays of biomarkers present in higher concentrations (CRP, RBP, and AGP) were designed in a competitive assay format. The competitor is a biotin-labeled version of each antigen that competes for capture antibody binding with the corresponding native antigen in the sample. The ferritin, HRP2, sTfR, and Tg assays were designed as sandwich immunoassays where secondary detector antibodies were included. The prototype kits contained the printed plates, sample diluent, lyophilized diluent additive containing the biotin-labeled antigen competitors (AGP, CRP, RBP), detection mix containing biotin-labeled secondary antibody (ferritin, HRP2, sTfR, and Tg), wash solution concentrate, streptavidin-horseradish peroxidase (SHRP) conjugate, and chemiluminescent substrate.

The calibrator reagent for sample quantification was prepared by combining each antigen at appropriate concentrations as originally defined by gravimetric methods to reflect the clinical range for each of the biomarkers used (Table 1). The relevant antigens were purchased from HyTest (Turku, Finland; RBP and CRP); Sigma-Aldrich (St. Louis, MI, USA; AGP); Bio-Rad Laboratories Inc. (Hercules, CA, USA; ferritin); US Biological (Salem, MA, USA; sTfR); BiosPacific Inc. (Emeryville, CA, USA; Tg); and CTK Biotech (San Diego, CA, USA; HRP2). Calibrators were added to a proprietary stabilization matrix, mixed, and lyophilized.

**Table 1:** Assay lower limits of detection (LLD), precision, and assay linearity for 7-Plex and conventional ELISAs.

	LLD			Intra-assay CV (%)		Inter-assay CV (%)		Linearity, mean (95% CI)
Analyte	7-Plex	Conv.^a^	Control	7-Plex	Conv.^a^	7-Plex	Conv.^b^	%
AGP (g/L)	0.0019	0.0039	High	7.1	4	9.6	0.5	105 (94–114)
			Medium	3.1	-^c^	5.3	-^c^	
			Low	2.6	6.2	7.3	7.1	
CRP (mg/L)	0.111	0.00015	High	6.1	2.7	6.5	9.9	95 (72–119)
			Medium	2.2	-^c^	6.4	^c^	
			Low	3.4	3.5	8.7	9.4	
Ferritin (μg/L)	0.206	0.59	High	2.1	4.7	8.4	8.7	100 (93–107)
			Medium	2.7	9.6	6.2	7.7	
			Low	3.8	5.8	9.7	6.8	
HRP2 (μg/L)	0.0017	0.0176	High	2.6	4.7	8.2	9.8	104 (82–127)
			Medium	5.3	5.3	12.3	17.9	
			Low	7	7.8	13.1	18	
RBP (μmol/L)	0.0054	0.052	High	3.9	6.7^d^	12.3	8.9^d^	92 (64–119)
			Medium	5.8	-	10.7	-	
			Low	4.9	-	10	-	
sTfR (mg/L)	0.192	0.036	High	3.9	6.2	12.4	5.7	117 (107–126)
			Medium	4.3	7.1	9.9	5.4	
			Low	4.3	4.3	13.9	6.4	
Tg (μg/L)	0.0244	0.44	High	3.1	6.6	8.1	5.7	103 (95–112)
			Medium	3.6	3.6	7.1	6.1	
			Low	3.7	6.6	13	9	

Control samples for the 7-Plex were High (1:5), Medium (1:10), or Low (1:20), diluted in sample buffer.

^a^ Conventional ELISA (Conv.) performance data as detailed in the product insert.

^b^ % CV across the dilution ranges.

^c^ Medium control values were not provided by the manufacturer.

^d^ For RBP, precision given as average intra- and inter-assay CVs in the calibrated range. CV, coefficient of variation; LLD, lower limit of detection; CL, confidence interval.

### USA donor training panel

A panel of 72 lithium heparin anticoagulated whole blood samples, 36 from males and 36 from females, was procured from Bioreclamation IVT Inc. (Westbury, NY, USA) and treated as previously described to prepare plasma fractions [5]. The plasma fractions were aliquoted into cryovials and stored at -80°C until use.

### NiMaNu cohort samples

Heparinized plasma samples from a study of MN status among pregnant women in Niger were used to evaluate the Human Micronutrient (7-Plex) technology [37]. Samples were collected as part of a cross-sectional study embedded into the Niger Maternal Nutrition (NiMaNu) Project, which was registered with the U.S. National Institutes of Health (www.ClinicalTrials.gov; NCT01832688). Ethical approval for the study protocol and the consent procedure was provided by the National Consultative Ethical Committee (Niger) and the Institutional Review Board of the University of California Davis (UC Davis; USA). Written informed consent, documented as a signature or a fingerprint in the presence of an impartial witness, was obtained from all subjects. Briefly, 18 rural health centers from 2 health districts in the Zinder Region were selected to participate in the NiMaNu project [41]. In each community, pregnant women were randomly selected and invited to participate in the survey. They were eligible if they provided written informed consent, had resided in the village for at least six months, and had no plans to move within the coming two months. As part of the NiMaNu study, heparinized plasma derived from venous blood samples were collected for ELISA measurement of AGP, CRP, ferritin, HRP2, RBP, and sTfR [37] and dried blood spots (DBS) cards were prepared from the venous blood samples and analyzed for Tg via a sandwich ELISA [28,41]. Plasma HRP2 concentrations in the plasma samples were measured using a commercially available CELISA kit (Cellabs Pty Ltd., Brookvale, Australia) [37].

A subset of 206 plasma specimens was selected from 654 original NiMaNu study specimens for which there was a remaining aliquot of plasma that had not been through a freeze-thaw cycle, with preference given to samples with original study data available for all seven analytes. The PATH and UW team used a blinded data file provided by the UC Davis team to select samples with the highest and lowest results for each of the seven analytes. This approach ensured that the resulting subset included specimens purposely identified to include a broad range of concentrations. UC Davis then provided the selected plasma specimens in tubes labeled with different identifiers so that the PATH and UW laboratories could remain blinded to the original NiMaNu study results until testing using the 7-Plex assay was completed.

### Human micronutrient (7-Plex) assay procedure

The lyophilized competitor mix was reconstituted into the entire sample diluent provided by the kit. Standard curves for each analyte were prepared by reconstituting the lyophilized calibrator with complete sample diluent/competitor mix and then as a further series of six threefold dilutions to create a seven-point dilution set in addition to a sample diluent–only negative control or blank (see Table 1 for upper and lower values). Next, each specimen (16 μL) was diluted 1:10 in complete sample diluent, with 50 μL of this added to the plate in duplicate wells. After addition of the standards and samples, each plate was incubated at room temperature with shaking. All plates were mixed using a flatbed shaker (Titertek–Berthold, Huntsville, AL, USA) at 500 rpm for 1 hour. All reactions were aspirated and washed 3 times with 400 μL of 1x Tris buffered saline Tween20 using an automatic plate washer (ELx50 plate washer, BioTek Instruments Inc., Winooski, VT, USA). Next, 50 μL of detection mix was added to each well and the plate was then incubated with shaking for an hour and washed one more time as described above. Labeling was performed by adding 50 μL streptavidin horseradish peroxidase solution to each well and shaking for 20 minutes, then the plate samples were aspirated and washed twice. The chemiluminescent substrate parts A and B were mixed in equal volumes and 50 μL of the mixture was then added to each well. Each plate was then imaged at 270 seconds of exposure time using the Q-View Imager LS (Quansys Biosciences). Q-View Software (Quansys Biosciences) was used to overlay a plate map onto the locations of analyte spots in each well and to measure the chemiluminescent signal from each spot in units of pixel intensity. The software applies the calibrator concentration values to the pixel intensities for each spot in the standard curve wells and fits 5 parameter logistic calibration curves for each analyte. The pixel intensities of the spots in each test well are then used to interpolate the concentration of each analyte relative to its calibrator curve. Once the plate image is overlaid with the analysis grid, all of the curve fitting and data reduction steps are automatically applied via the software. The upper and lower limits of detection were calculated using the highest and lowest concentration of each analyte in the calibrator.

### Conventional assay methodologies used with the USA donor training panel

The concentrations of AGP, CRP, Ferritin, RBP, sTfR and Tg were established using ELISA kits for each biomarker. These were the reference methods to which the new 7-plex method would be compared (Table 1). As the donor panel was derived from a USA-based population there is no exposure to P. *falciparum* malaria and so HRP2 measurement was not performed. Where possible, preference was given to assays widely used in previous studies of MN status. Commercial ELISA kits were obtained for the following biomarkers: Tg (DRG International, Springfield Township, NJ, USA); sTfR (R&D Systems Inc., Minneapolis, MN, USA); ferritin (Ramco Laboratories, Inc., Houston, TX, USA); RBP (Scimedx, Denville, NJ, USA); and AGP (GenWay Biotech, San Diego, CA, USA). The values for CRP were obtained through a previously described in-house ELISA method [42]. With the commercial kits, the USA donor training panel samples were processed and data analyzed according to each manufacturer’s instructions for use. All conventional assay results were obtained using a Synergy HT plate reader (BioTek Instruments Inc., Winooski, VT, USA). Qualified standard materials were purchased from National Institute for Biological Standards and Control (NIBSC, Potters Bar, UK) and Sigma-Aldrich (Table 2) and serial dilutions screened using both the conventional ELISAs and the 7-Plex plate.

**Table 2:** 7-Plex assay calibration ranges and recovery via comparisons with conventional ELISAs from USA donor training panel volunteers.

	AGP(g/L)	CRP (mg/L)	Ferritin (μg/L)	HRP2 (μg/L)	RBP4 (μmol/L)	sTfR (mg/L)	Tg(μg/L)
**Calibration**							
Gravimetric calibrator range	0.003–1.9	0.1–62.0	0.22–160	0.01–6.95	0.005–3.43	0.02–11.8	0.06–45.0
Referenced calibrator range	0.004–2.9	0.081–59.0	1.3–981.2	N/A	N/A	4.0–2890.0	0.29–210.0
Reference source	ERM-DA470K/IFCC	NIBSC 85/506	Harmonized to RAMCO kit	Gravimetric	Gravimetric	NIBSC 07/202	CRM-457
**Recovery**							
n (pairs)	72	71	65^a^	-	72	72	68^a^
Gravimetric calibrator values	81% (15%)	152% (110%)	19% (9%)	-	114% (20%)	1.5% (0.4%)	29% (13%)
Referenced calibrator values	125% (23%)	145% (104%)	116% (58%)	NA^b^	NA	373% (108%)	136% (61%)
**Spearman Correlations**^c^							
	0.824	0.962	0.894	NA^b^	0.781	0.723	0.866

The Spearman correlation for the 7-Plex and respective conventional assays is also shown. The mean (SD) recovery for 72 heparin plasma specimens was assessed using both gravimetric and referenced calibrator values, with 7-Plex assay value expressed as a percentage of conventional kit value. The Spearman Correlations were derived from 7-Plex and conventional immunoassay data.

^a^ For ferritin, seven samples were below the detection limit for the conventional assay; similarly with Tg, four samples with results below the detection limit for the DRG assay kit.

^b^ HRP2 data not included in pairwise comparisons or Spearman correlation, as all 72 samples were negative for parasitemia.

^c^ All Spearman correlations were significant at the 0.01 level (2-tailed).

### Statistical methods

#### Human micronutrient (7-Plex) assay performance

Performance for each analyte assay in the panel was assessed by a number of standard validation experiments. For the 7-Plex assay precision, linearity, and specificity were determined by the following standard validation experiments. Inter- and intra-assay precision were estimated using low, medium, and high controls assayed on 14 plates from 3 production batches to calculate coefficients of variation using a variance components model. Linearity was assessed by assay of 5 plasma samples diluted at 1:5, 1:10, 1:20, 1:40 and 1:80, and was calculated by dividing the concentration of a diluted sample by the concentration of the next higher dilution, multiplying by 2, and expressing as a percentage. Specificity was assessed by testing antigens and detection reagents individually using a multiplexed plate. Percent cross-reactivity was calculated by dividing the calculated concentration of the antigen with its detection into the calculated concentration of other antigens and detections on that spot. The assay upper limit of detection used the highest concentration of the analyte calibrators and the lower limit of detection (LLD) was the concentration of the lowest dilution of the calibrators.

#### Referencing calibrator concentrations

Where available, reference materials certified by the World Health Organization or European Commission Joint Research Centre were measured by the 7-Plex and conventional assays. Results were evaluated by dividing the observed result by the expected certified value. The concentration of the ferritin assay calibrators was reassigned based on values calculated from the conventional assay concentration, giving improved harmonization as compared to the initial value defined by gravimetric preparation (Table 1).

#### Comparative analyses of test datasets

The results from the conventional ELISAs, and the 7-Plex for the USA donor training panel (72 plasma specimens) were compared by a variety of methods for all biomarkers (except HRP2, which is absent in the USA donor training panel). Similarly the same methodology was used with the NiMaNu panel (206 plasma specimens) to compare the 7-plex data to the original data derived from the VitMin Lab (in-house ELISAs to AGP, CRP, ferritin, RBP and sTfR [36]), in-house testing (Tg) and a commercial ELISA (HRP2). Comparisons by scatter plots and Spearman correlation were used to evaluate whether results from the assays covaried linearly. Absolute levels were compared by expressing results from the 7-Plex assay as a percentage of the results from conventional assays, and concentration-dependent bias in differences between measures from the 7-Plex assay and original study assays were evaluated using Bland-Altman plots with the average concentration value for the two methods (x-axes) plotted against the percent difference ([7-Plex result–conventional result)/ average concentration] *100) on the y-axes [43,44].

#### Sensitivity and specificity versus the NiMaNu panel data as derived from receiver operating characteristic curves

We evaluated the use of analytical measurement from the 7-Plex assay to correctly identify cases of deficiency, inflammation, or infection by calculating diagnostic sensitivity and specificity using results of the NiMaNu panel as a gold-standard. Receiver operating characteristic (ROC) curves plotting sensitivity (y-axis) against specificity (x-axis) were generated to identify optimal threshold values for each analyte as measured in the 7-Plex. Optimal threshold values were defined as those resulting in maximal area under the ROC curves (AUC), with the line of equality (area under the curve of 0.5) indicating the expected result for random categorization, and an area under the curve of 1.0 indicating perfect predictive value. Determination of positive or negative for deficiency, inflammation, or infection for the NiMaNu assay results relied upon threshold values used in that study. Thresholds to define inflammation were as follows: CRP ≥5 mg/dL and AGP ≥1 g/L; and to define deficiency: RBP <14.7 μg/mL, sTfR >8.3 μg/mL, and ferritin <15 μg/L. For Tg, there is no universal threshold to indicate iodine deficiency in pregnant women, however majority of studies report that iodine-deficient pregnant women have a median Tg ≥13 μg/L [45]. A value of 13 μg/L was set as the threshold for the NiMaNu Tg assay results solely for the purposes of comparing the 7-Plex assay and conventional assays, however, a recent study has noted that when using DBS, a threshold of Tg ≥10 μg/L is proposed [26,27]. With HRP2, different core analysis methods were used, the NiMaNu panel used an immunoassay that measured optical density whilst the 7-plex HRP2 assay used pixel intensity. Therefore sensitivity and specificity for HRP2 was assessed using the diagnostic outcome, i.e. malaria positive or negative.

## Results and discussion

### 7-Plex assay development

The 7-Plex assay performance data are summarized and compared to the conventional assay parameters in Table 1. Data derived from 14 independent 7-Plex assay plates were used to establish the precision (intra- and inter-assay, n = 4 samples) and linearity (five dilutions of n = 6 samples) for each assay, and to confirm the LLD for each assay. Results were compared to performance data provided in the product inserts of the conventional ELISAs used to quantify the same biomarkers (Table 1). The LLD of the biomarkers used on the 7-Plex assay was significantly more sensitive for HRP2, RBP, and Tg while similar in some instances with the conventional assays (e.g. AGP, ferritin). sTfR had a higher LLD for the 7-Plex assay as compared to the conventional assay (0.192 mg/L versus 0.036 mg/L). The CRP assay was significantly less sensitive than the conventional assay as the latter was developed to be highly sensitive with very dilute samples [42]. Despite this, the 7-plex CRP assay has a LLD that is more than one log greater than the threshold to indicate inflammation [46]. All of these LLDs, as derived from the 7-Plex are below the respective cut-off values for indicating an MN deficiency or inflammation [26,36,47,48]. The specificity, sensitivity, precision, and linearity results met required specifications for all analytes. 7-Plex inter- and intra-assay CVs were under 15% (Table 1) which is an accepted maximum for inter-and intra-assay CVs for ELISAs [49], and was comparable to precision reported for the conventional ELISA kits. Tests across dilution series from 1:5 to 1:80 found 95% confidence intervals including the acceptable range of 90–110% linearity for all analytes with the exception of sTfR (117%).

The concentration of each calibrator was initially assigned by calculation based on the volume of stock solutions (of a known concentration) of each biomarker added to the calibrator material (measured gravimetrically). The calibrator concentrations were later adjusted via qualified reference standards, where possible, to better standardize the performance of each assay (Table 2).

The performance of the 7-Plex assay was then assessed on the training panel of heparinized plasma collected from a cohort of 72 US adults (Table 2) [5]. To improve assay performance, the biomarker concentrations in the 7-Plex assay were established via reassigned calibrator values based upon international reference standards (where possible), gravimetric measuring (HRP2 and RBP), or harmonized to another conventional ELISA method (e.g., ferritin, Table 2). The harmonization method used here was applied because the NIBSC referencing for ferritin still gave poor agreement with both gravimetric measurement and established assays [50].

Summary statistics from the 7-Plex assays and conventional assays from both the training panel and the NiMaNu study specimens are shown in S1 Table. Average concentrations and ranges suggest the expected differences in inflammation and deficiency by population and differed between assay methods for some analytes, as described in detail below. Spearman correlations showed strong association between results for the USA donor training panel tested with the 7-Plex and conventional immunoassays (Table 2). Strong associations are also evident in linear regression plots (S1 Fig) but with some large differences in absolute value for sTfR observed in the training panel. Bland-Altman analyses (S2 Fig) show the magnitude of differences and concentration-dependent bias between the 7-Plex and conventional immunoassays for each analyte. 7-Plex assay results were higher than conventional assay results for all analytes. While mean percent difference was large for some analytes (up to 111% mean difference for sTfR), differences were consistent across the concentration ranges, with few results outside the 95% confidence interval. Differences in absolute values, as assessed by expressing 7-Plex assay results as a percent of conventional immunoassay results, are shown in Table 2.

Reference materials measured in the 7-Plex and conventional immunoassays as compared to their certified values are shown in Table 2. Conventional assays showed good recovery of the expected values for certified reference materials tested at a range of dilutions. For AGP, CRP, sTfR and Tg, the 7-Plex assays also showed improved recovery of certified values, reflecting the calibrator referencing to these materials. No certified reference materials were available for RBP and HRP2; therefore, the gravimetric references for RBP and HRP2 were retained. The 7-Plex ferritin assay was originally referenced to NIBSC 94–572, and testing of that reference material at 4 dilutions found measured values averaging 7776 ± 1752 μg/L, 123% of the certified value of 6300 μg/L. However, referencing the 7-Plex calibrator to NIBSC 94–572 resulted in recovery averaging only 12% ± 5% of ferritin concentrations as measured by the Ramco ferritin assay for the USA donor training panel.

Using a method similar to inter-method harmonization, as described by Wilson, et al. [50], we modeled the relationship between pixel intensity observed in the 7-Plex ferritin assays and the concentrations derived from the Ramco ferritin assay for the set of 72 plasma specimens. Data were fit to a second order polynomial model. These results were then used to estimate a new concentration value for the mid-point of the 7-Plex ferritin standard curve based on pixel intensity for that standard point averaged across 8 plates. Applying this new calibrator referencing method significantly improved recovery for the USA donor training panel specimens (127% ± 52%), but using this method also results in significant over-recovery of the certified concentration of NIBSC 94–572 (1346% ± 303%).

### Analysis of the NiMaNu panel derived from a cohort of pregnant women in Niger

Spearman correlations between the 7-Plex assays and original study results were high (0.840–0.987) for plasma AGP, CRP, ferritin, RBP and sTfR concentrations, and moderate for Tg (0.753) which compared 7-Plex plasma results to NiMaNu DBS results (Table 3). HRP2 optical density measures from the NiMaNu study were moderately correlated (0.553) to pixel intensity in the 7-Plex HRP2 assay. Recovery results showed acceptable agreement between assay methods for AGP and RBP, and higher variability in recovery for CRP, ferritin, and sTfR but significant differences in absolute values for Tg (Table 3). Summary statistics from the 7-Plex assays and conventional assays from the NiMaNu study specimens are in S1 Table.

**Table 3:** Spearman correlations and recovery, 7-Plex assays vs NiMaNu data.

	AGP(g/L)	CRP (mg/L)	Ferritin (μg/L)	HRP2 (μg/L)	RBP4 (μmol/L)	sTfR (mg/L)	Tg(μg/L)
n	206	204^a^	203^a^	206	206	206	190^b^
Spearman correlation ^c^	0.900	0.987	0.950	0.553^d^	0.872	0.840	0.783^c^
7-Plex/NiMaNu mean ± SD (%)	100± 28	75± 53	122± 84	-	92± 16	150± 49	49± 18^c^
Sensitivity and specificity							
NiMaNu Threshold	>1.00	>5.0	<15.0	NA	<1.32	>8.3	>13.0^e^
7-Plex Optimal Threshold	>0.67	>3.3	<16.8	>0.92	<1.20	>11.7	>7.2
7-Plex Sensitivity (%)	74	91	88	93	87	86	89
7-Plex Specificity (%)	90	95	90	98	83	81	85

^a^ Samples below assay detection limits for either the 7-Plex or NiMaNu immunoassays were excluded.

^b^ Nine samples with extraordinarily high Tg results in the initial NiMaNu dataset were excluded from analyses. An additional 7 samples were not tested for Tg in the NiMaNu study [41] and so no comparison could be made.

^c^ NiMaNu Tg measured in dried blood spots versus 7-Plex Tg measured in lithium heparin plasma.

^d^ HRP2 data compares NiMaNu optical density results to 7-Plex pixel intensities and concentration data were not available for the NiMaNu dataset.

^e^ The majority of studies typically report that iodine-deficient pregnant women have a median Tg ≥13 μg/L and so used here solely for the purposes of comparing assay methods [45].

A total of 1442 data points for the 7 biomarkers from the 206 NiMaNu samples were generated by the 7-Plex assay. From the initial 7-Plex assay, there were only 21 test results that were out of range (upper or lower) and only 4 samples that failed to meet quality control (QC) criteria of an intra-assay CV of <15% for duplicate sample wells. Only 3 assays were affected by being out of range limits: CRP (n = 12; ten upper, 2 lower), ferritin (n = 8; 7 upper, 1 lower), and sTfR (1 lower). Some samples were out of range for more than one analyte, meaning that retesting a single sample permitted measurement of more than one biomarker that was out of range. Therefore, only 25 from 1442 test results (1.7% of the entire test panel) had to be reassayed.

**Fig 2:**
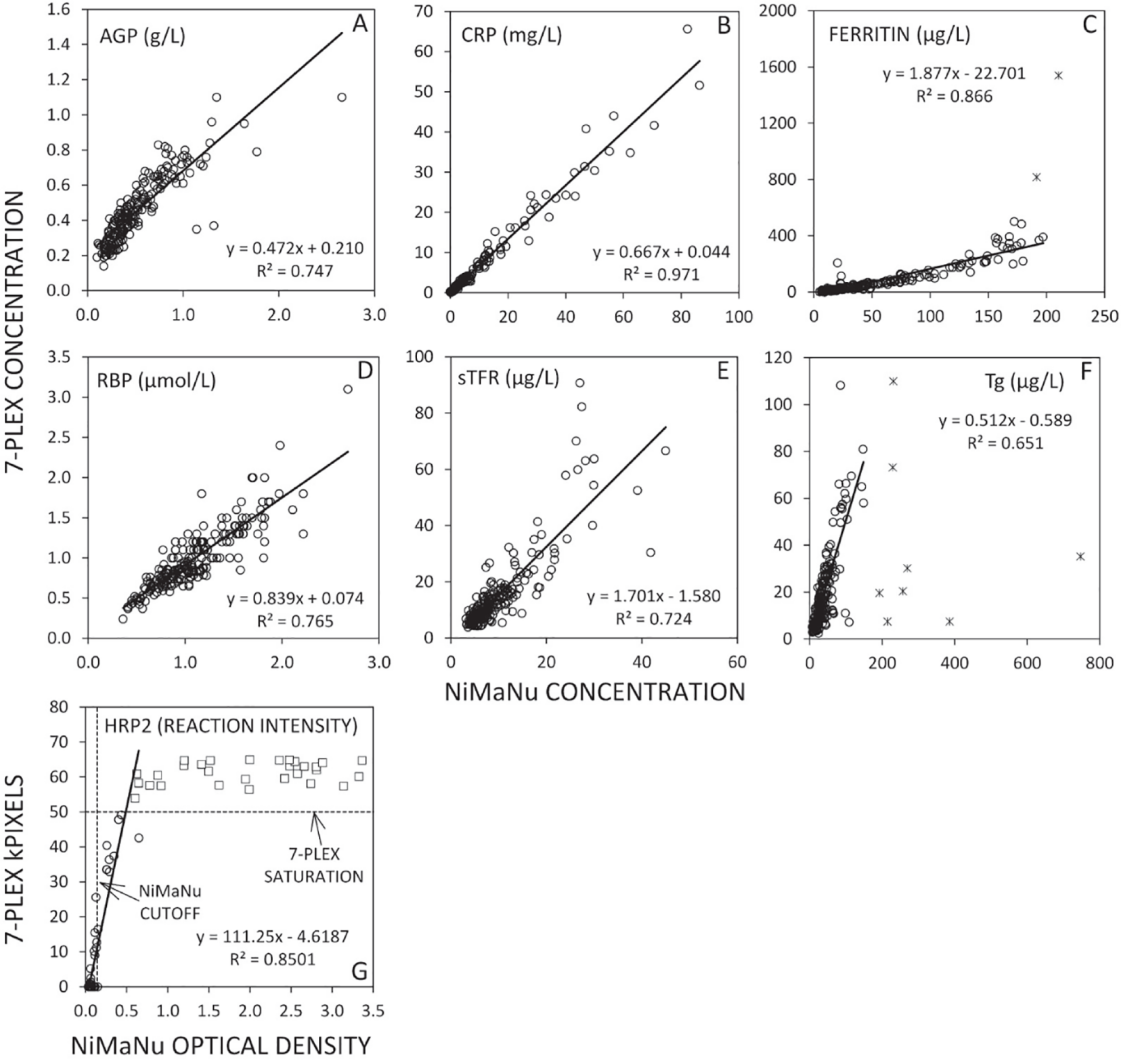
Scatter plots of the 7-Plex results versus NiMaNu conventional immunoassay results. Concentrations of each analyte as measured in the 7-Plex (x-axes) plotted against concentrations measured using conventional assays (y-axes) for the NiMaNu panel. Solid line is linear least squares regression (y = mx+b). For ferritin, 2 outliers were excluded from regression and for Tg, 9 outliers were excluded from regression (the outliers are included in the plots marked as x rather open circles); 3 extremely high NiMaNu Tg values excluded from plot. For HRP2, the plot reflects assay signal intensity rather than concentration as concentration was not available from the NiMaNu dataset. Horizontal dotted line indicates cutoff for positive results based on the conventional immunoassay and vertical line indicates 7-Plex assay results beyond assay saturation. AGP, α-1-acid glycoprotein; CRP, C-reactive protein; HRP2, histidine rich protein II; RBP, retinol binding protein 4; sTfR, soluble transferrin receptor; Tg, thyroglobulin.

**Fig 3:**
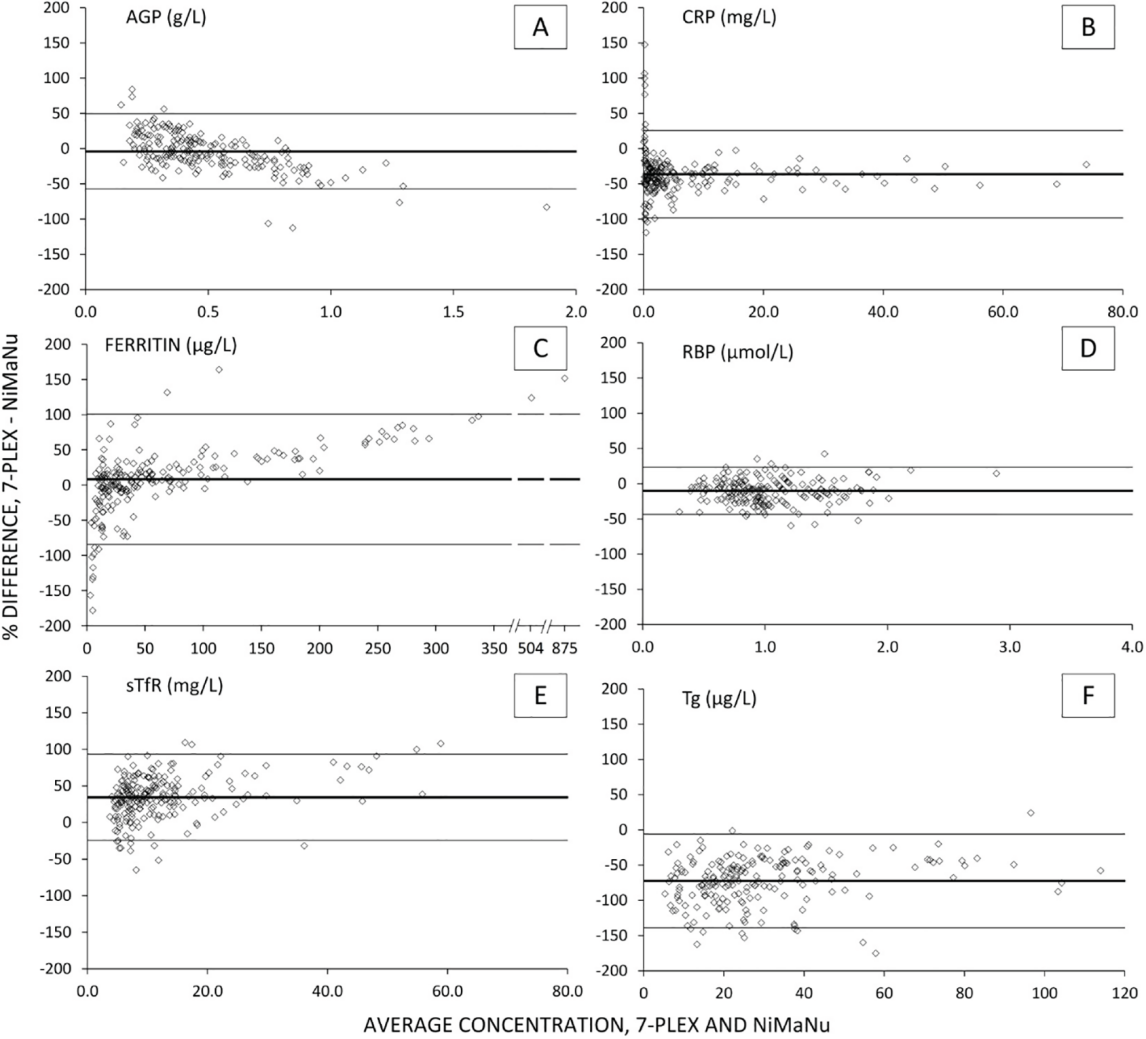
Bland Altman plots, 7-Plex vs NiMaNu immunoassay results. Bland-Altman plots showing percent difference between the 7-Plex and the NiMaNu conventional immunoassay results on the y-axes plotted against average concentration on the x-axes. Heavy horizontal line and light horizontal lines indicate mean ± 2 standard deviations of percent difference. AGP, α-1-acid glycoprotein; CRP, C-reactive protein; RBP, retinol binding protein 4; sTfR, soluble transferrin receptor; Tg, thyroglobulin.

Further comparison of the data sets was made via scatter plots and Bland Altman analyses (Figs 2 and 3, respectively). The scatter plots for ferritin had two data points removed and nine data points were removed for Tg as these outliers grossly affected the pooled data (Fig 2). Each plot reflects the generally good agreement between methods, particularly within concentration ranges necessary for identifying deficiency or inflammation. The Bland Altman plots (Fig 3) did not show significant concentration dependent bias and showed the greatest differences between assays when values were at either extreme of the concentration range. The CRP and ferritin Bland Altman plots, in particular, show significant variability at very low concentrations, which contributes to the overall variability seen in recovery results, but does not reflect physiologically meaningful differences in concentration (e.g. average values less than 0.5 mg/L for CRP, Fig 3). With regard to Tg, where the greatest bias is observed, the original analyses of the NiMaNu samples was using DBS as the sample type while the 7-Plex assay used plasma and so we suspect that the differences in specimen types or matrix may be reflected in the differences in recovery.

Our initial evaluation of the panel showed significant differences in absolute values between results from an R&D Systems sTfR ELISA kit and the 7-Plex sTfR (Table 2). The NiMaNu specimens were also assayed using the R&D Systems sTfR ELISA. While Spearman correlations between the 7-Plex, the R&D kit, and the sTfR assay used for the NiMaNu study were generally high (0.840 to 0.969), the 3 assays varied in absolute values, with both the 7-Plex and NiMaNu sTfR assay (performed by the VitMin Lab, Willstaett, Germany) yielding significantly higher concentrations than the R&D Systems kit (see S2 Table). This is consistent with the challenges previously observed with harmonizing sTfR recovery values via different immunoassay methods [51,52].

For Tg, the Spearman correlation between the 7-Plex and revised NiMaNu results was originally 0.753 (n = 199). Scatterplots (Fig 2) show nine samples for which Tg values from the original study were exceptionally high and their agreement with the 7-Plex results was poor. Boxplots and stem and leaf plots of log transformed Tg results from the NiMaNu study confirmed these 9 specimens as outliers (as confirmed by boxplots cutoff definition of +/- 1.5 x interquartile range of the data). When these outliers are excluded, Spearman correlation between 7-Plex and NiMaNu data improves slightly to 0.783 (n = 190; Table 3). To further investigate the discrepancies, the 9 samples and a randomly selected set of 23 other specimens were retested using the DRG ELISA assay. The re-analysis suggested these nine outliers are anomalous results from the NiMaNu set. There was good agreement between the 7-Plex and the DRG kit when all 32 samples were included (Spearman correlation, 0.877), and lower correlation between the NiMaNu results and DRG kit (Spearman correlation, 0.604). When the 9 outliers as measured in the NiMaNu study are excluded, correlation is improved across all three assay methods for the remaining 23 specimens (7-Plex vs NiMaNu, 0.770 and DRG kit vs NiMaNu, 0.824).

Sensitivity and specificity analysis was used to determine whether the data derived via the 7-Plex could be used correctly to categorize individual samples as deficient/sufficient (ferritin, RBP, sTfR, and Tg), positive or negative for malaria parasitemia (HRP2), and positive or negative for acute inflammation (AGP and CRP), using the classification from the NiMaNu study as the gold standard (Table 3, [37]). ROC curve analyses were used to identify the optimal threshold value to apply for each assay hosted on the 7-Plex, with equal weight given to sensitivity and specificity (S3 Fig). Using the optimal threshold for each analyte, sensitivity ranged from 74% (AGP) to 93% (HRP2) and specificity ranged from 81% (sTfR) to 98% (HRP2; Table 3).

This work was designed to expand upon a pre-existing multiplexed assay that we have previously developed by adding additional biomarkers for MN status assessment, and to make improvements to the original assays included in the Quansys Bioscience Q-Plex™ technology [5]. New assays were added to the array to interrogate for Tg as an indicator of iodine deficiency and an HRP2 assay to indicate current or recent infection by *P*. *falciparum*. The sTfR assay was redesigned because an antibody used in the original design was no longer available. All of the assays were successfully integrated into an immuno-array and then further optimized to allow a single dilution of small sample volume for the simultaneous quantification of seven analytes using a traditional ELISA protocol and equipment, apart from the Q-viewer for plate reading and analysis. In addition, a reference spot was also added to the array in order to simplify the alignment of the data capture grid of the analysis tool with each well of the 96 well plate image. The Q-View software automatically fits calibration curves for all seven analytes using a log 5-parameter logistic model, thus simplifying analysis, as compared to conventional assays which may require the use of additional software to fit assay calibration curves and calculate concentration. Verification experiments demonstrated that the resulting 7-Plex panel met performance requirements for assay precision, linearity, and limits of detection. Good correlation, sensitivity and specificity in comparisons to results of conventional assay results for samples collected from a population at risk for deficiency, inflammation and malaria parasitemia suggest the 7-Plex assay offers a promising alternative to existing methods.

In our previous study, we used the Bio-Rad Liquichek 3 control as the calibrator for our reference curve values [5,36]. While acceptable, such quality control materials are derived from pooled human serum samples, and as such, the range of each biomarker to be interrogated is variable and may not be of the appropriate concentration necessary to generate a standard curve that reflects the clinical range required. By procuring purified antigens from vendors and spiking these into a serum-analog matrix, we were able to develop a calibrator that is less prone to batch-to-batch variance and importantly the reference range for each biomarker’s calibrator can be more precisely determined to allow the cut off value for each biomarker to be established and to remain constant towards the mid-range of the standard curve (where possible). The use of international reference standards (AGP, CRP, ferritin, Tg, and sTfR) and a training panel, previously developed by our group [5], provided the opportunity to more accurately define the calibrator concentrations and enable more accurate quantification of the biomarkers, as compared to other assays that detect the same MN biomarkers.

A lack of harmonization between different ELISAs to the same target biomarker is commonly observed and their standardization remains a challenge [50,53]. Our development reflects also this where the 7-Plex and conventional immunoassay methods show excellent correlation but some analytes showed vastly different absolute values between methods. Two of the analytes, ferritin and sTfR, were notable for their difference in absolute values and thus warranted further investigation.

We noted that the ferritin results differed significantly from the Ramco kit results when the 7-Plex calibrator was referenced to the NIBSC standard, with recovery averaging 12%. The NIBSC standard was intended to qualify the standard concentration but we found that it was poor when used with our assay. The NIBSC ferritin standard is a recombinant protein expressed from a bacterial source and therefore it may be possible that some epitopes are absent or altered in this molecule as compared to human ferritin. We hypothesize that one or both of the monoclonal antibodies used in the 7-Plex sandwich ELISA has reduced avidity to the ferritin standard used. However, an inter-laboratory evaluation of this standard showed generally good high concordance with the standard values [51]. We then chose to follow the approach described by Wilson [50] to harmonize concentration values to the curve based on ferritin values assigned by the Ramco assay and to use those results to re-reference the 7-Plex calibrator. Furthermore, applying harmonized calibrator values for 7-Plex testing of the NiMaNu panel shows that this harmonization approach yields generally good agreement in absolute values across the Ramco, 7-Plex and ferritin assays conducted for the NiMaNu study.

Similarly, the sTfR results were significantly different in the 7-Plex immunoassay versus the reference method (R&D or VitMin Labs). The challenges with standardization of sTfR immunoassays have been noted before with bias across methods prior to a reference standard being available; the NIBSC standard also uses a recombinant antigen [54]. Even with the reference standard now available, commercial sTfR assays used in different laboratories show significant variation in the measurement of this standard [13]. While NIBSC sTfR referencing results in higher values for the 7-Plex than for the R&D Systems assay kit, our evaluation of the NiMaNu cohort found that it results in generally good agreement with the sTfR ranges obtained using the method described by Erhardt, et al. [36].

A key limitation of our previous study was that the panel used to verify the original 5-plex assay was derived from an adult US cohort, which does not represent the typical populations that the tool is designed to survey in terms of more variable MN status and increased exposure to disease and/or malaria. In this study, we also demonstrate the performance of the 7-Plex array on samples derived from pregnant women in Niger. This represents a key population group where surveillance can play a role in understanding MN status in populations at risk. This panel was ideal for the initial verification of the 7-Plex as the same biomarkers had all been qualified by independent methods, with the exception of seven data points for Tg, from samples that were not analyzed. Overall, the assays on the 7-Plex array had good concordance with the predicate ELISAs when analyzed by Spearman correlation, and scatter plots, and Bland Altman analysis reflect overall agreement in spite of some cases of systematic difference in absolute value. The Bland Altman analyses were key to highlighting that some highly discordant test data points were at either very low or high levels, and so while significantly different, they present the same test outcome (i.e., a deficient or sufficient result would be derived from the same sample irrespective of the exact value recovered). This was further confirmed by applying ROC analyses to determine the optimal cut-off values that still gave high sensitivity and specificity of the 7-Plex assay as compared to the NiMaNu cut off threshold to establish MN status as sufficient or deficient, inflammation status, or malarial infection. When considering all the biomarkers assessed from the NiMaNu panel, only 1.7% had to be retested due to being out of range or being where duplicate test results were variant by >15%, indicating that the combination of broad calibrator ranges and the sample dilution volume permit the detection of most biomarkers in one test run. This limits the need to run multiple repeat assays using different sample dilutions in order to acquire a complete data set saving money, reducing the need for extra sample volumes and the time to process discrepant samples.

AGP had the poorest sensitivity in the NiMaNu samples of pregnant women via this analysis and this is largely in part to the few samples that had significantly elevated AGP levels with the majority of samples being tightly clustered at low concentrations. We hypothesize that AGP is prone to more significant variation within small sample dilutions as compared to the other biomarkers due to its relatively high concentration. For example, many conventional ELISA assays for AGP recommend a sample dilution of 1:10000 to prior to measurement of AGP, whereas the 7-Plex plasma sample volume is only diluted by 1:10. In some cases, the values for recovery were significantly different from the original assay while being concordant via Spearman and Bland Altman analysis.

While MN deficiencies affect vulnerable population groups globally, LMICs are most significantly affected. Most LMICs attempt to address these deficiencies via supplementation, fortification, and dietary and/or agricultural interventions to improve micronutrient intakes. However, the challenge in assessing MN status at the population level has led to large data gaps in terms of general surveillance. This is due to a variety of reasons but primarily because MN measurements are costly, difficult and complex to perform in-country based upon the current methodologies. Our tool is specifically targeted toward users in LMICs, with the intent of developing a practical and relatively low-cost tool (less than half that of conventional assays excluding other labor and consumables savings) to enable in-country surveillance of nutritional status across key populations and to measure the performance of ongoing nutritional interventions. We have developed a tool that can be used in a laboratory setting, does not need a technically complex reader, and requires only common laboratory equipment (e.g., a plate shaker and plate washer). The protocol follows a standard methodology for performing an ELISA assay and one trained laboratorian can process 10 plates in an 8-hour period (400 samples and therefore 2,800 data points for the 7 biomarkers on the array).

Further validation of the Human Micronutrient 7-Plex assay on other appropriate cohorts is necessary and is presently underway by our team and by other independent groups. We are currently investigating the use of dried blood or plasma spots as a sample type given that an assay only requires 16 μL of sample. We are also in the process of investigating the expansion of the range of assays hosted by the platform in order to assess other key MNs including folate, vitamin B12, and vitamin D.

## Supporting information

S1 Fig Scatter plots, 7-Plex versus conventional immunoassay results for plasma samples from USA donor panel. Concentrations of each analyte as measured in the 7-Plex (x-axes) plotted against concentrations measured using conventional assays (y-axes) for 72 lithium heparin plasma specimens. Solid line is linear regression. AGP, α-1-acid glycoprotein; CRP, C-reactive protein; RBP, retinol binding protein 4; sTfR, soluble transferrin receptor; Tg, thyroglobulin.
(TIF)

S2 Fig Bland-Altman analysis, 7-Plex results versus conventional immunoassay results for plasma samples from USA donor panel. Bland-Altman plots showing percent difference between 7-Plex and conventional assay results on the y-axes plotted against average concentration on the x-axes for 72 lithium heparin plasma specimens. Heavy horizontal line and light horizontal lines indicate mean ± 2 standard deviations of percent difference.AGP, α-1-acid glycoprotein; CRP, C-reactive protein; RBP, retinol binding protein 4; sTfR, soluble transferrin receptor; Tg, thyroglobulin.
(TIF)

S3 Fig Sensitivity and specificity of 7-Plex assays using NiMaNu panel data. Receiver operating characteristic (ROC) curves plotting sensitivity (y-axis) against 1-specificity (x-axis) were generated from 7-Plex results of pregnant women participating in NiMaNu study to identify optimal threshold values for each analyte as measured in the 7-Plex to be used in specificity and sensitivity analysis. Optimal threshold values, defined as those resulting in maximal area under the ROC curves (AUC), are reported in Table 3.
(TIF)

S1 Table Summary statistics from the 7-Plex and conventional assays from USA donor training Panel and NiMaNu panel. (DOCX)

S2 Table Performance comparison of different sTfR assays on both panels from USA donors and NiMaNu. Median concentrations and interquartile range (IQR) of sTfR as measured by three immunoassay methods per sample set and comparisons between assays in absolute value (% recovery) and by Spearman correlation.
(DOCX)
