# Supplementary figures and images for: Simultaneous assessment of iodine, iron, vitamin A, malarial antigenemia, and inflammation status biomarkers via a multiplex immunoassay method on a population of pregnant women from Niger
Source: PLoS One. 2017 Oct 5;12(10):e0185868. doi: 10.1371/journal.pone.0185868 (PMC5628875; doi:10.1371/journal.pone.0185868)

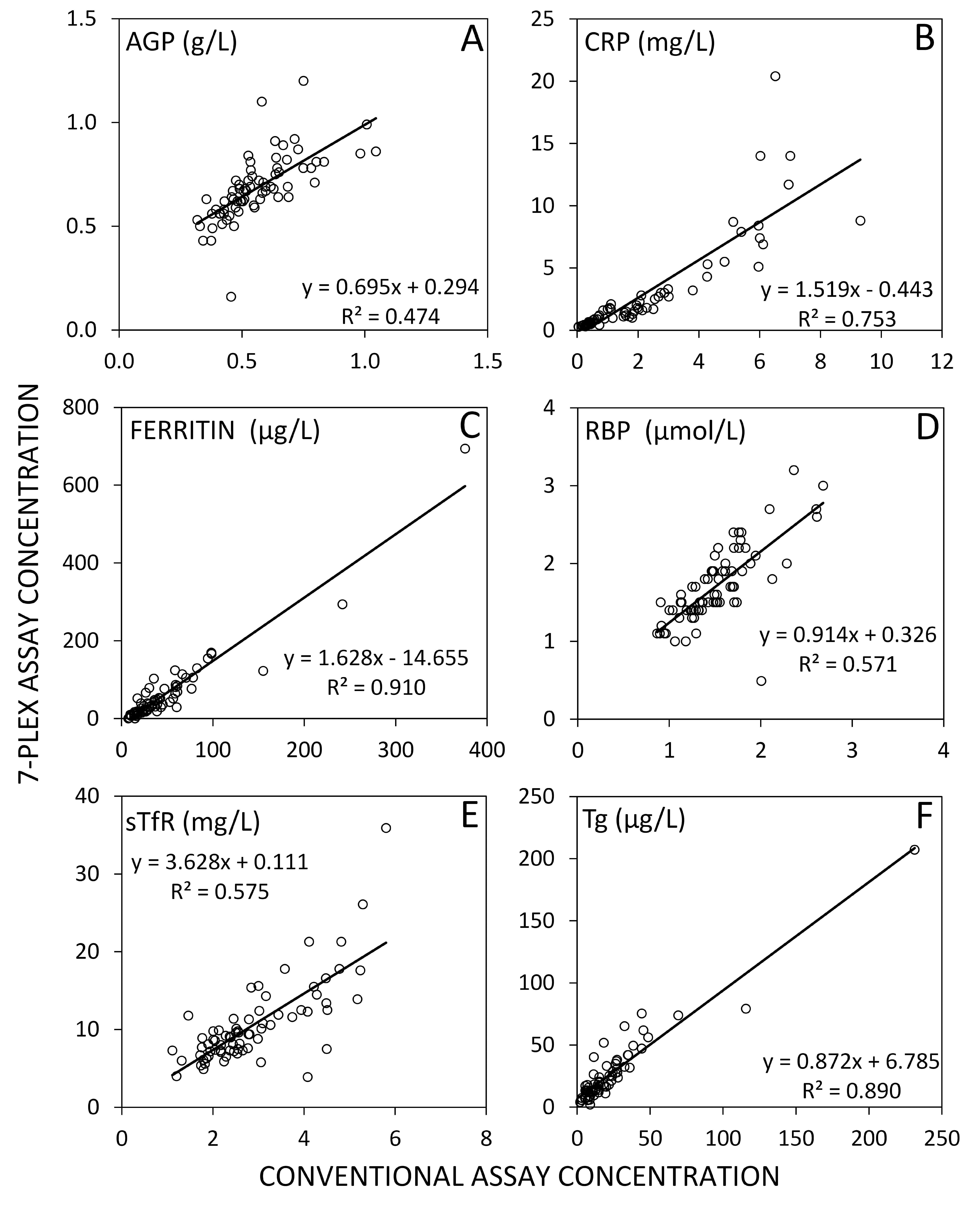

Supplement: S1 Fig — Concentrations of each analyte as measured in the 7-Plex (x-axes) plotted against concentrations measured using conventional assays (y-axes) for 72 lithium heparin plasma specimens. Solid line is linear regression. AGP, α-1-acid glycoprotein; CRP, C-reactive protein; RBP, retinol binding protein 4; sTfR, soluble transferrin receptor; Tg, thyroglobulin. (TIF) [file pone.0185868.s001.tif]

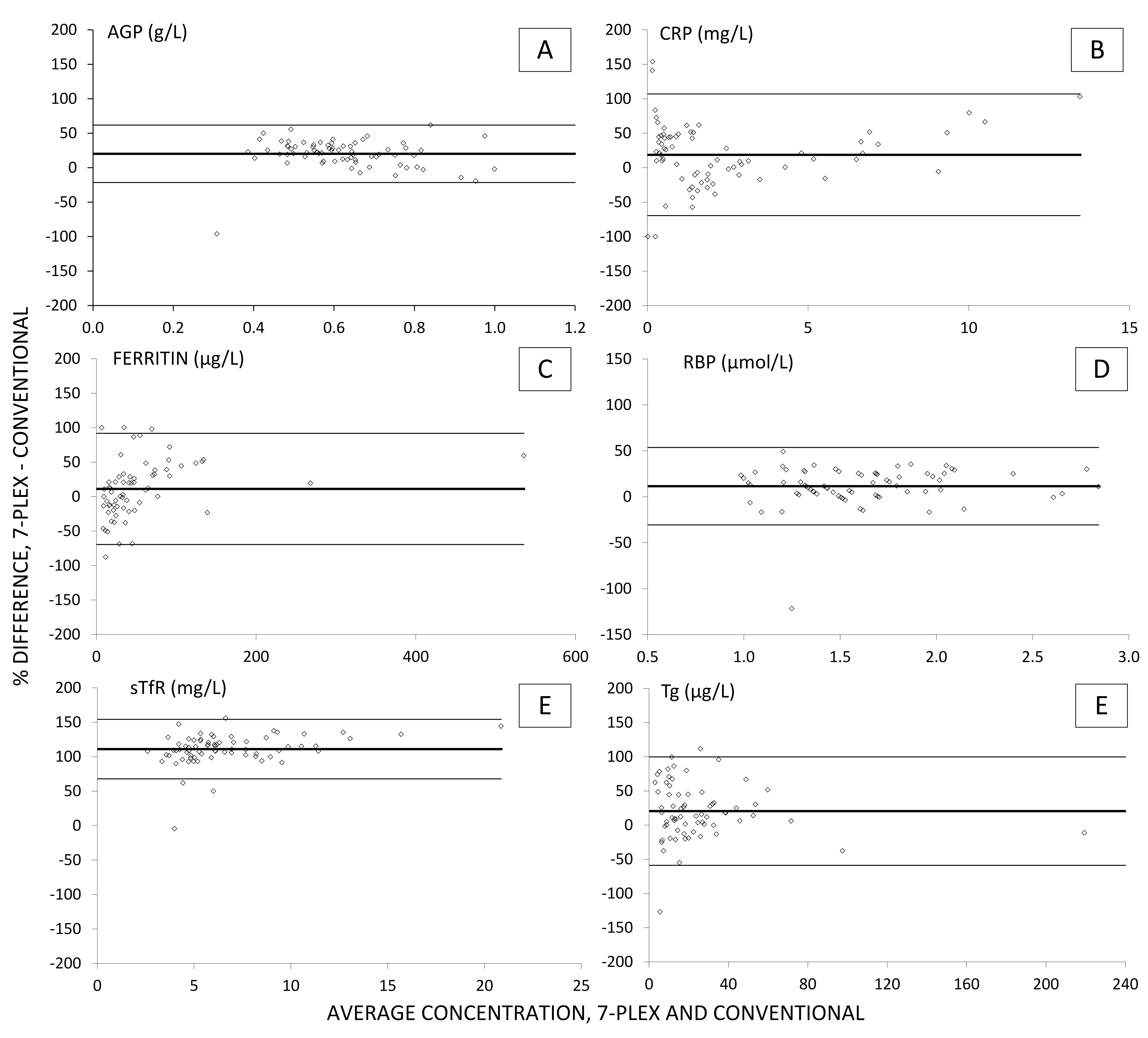

Supplement: S2 Fig — Bland-Altman plots showing percent difference between 7-Plex and conventional assay results on the y-axes plotted against average concentration on the x-axes for 72 lithium heparin plasma specimens. Heavy horizontal line and light horizontal lines indicate mean ± 2 standard deviations of percent difference.AGP, α-1-acid glycoprotein; CRP, C-reactive protein; RBP, retinol binding protein 4; sTfR, soluble transferrin receptor; Tg, thyroglobulin. (TIF) [file pone.0185868.s002.tif]

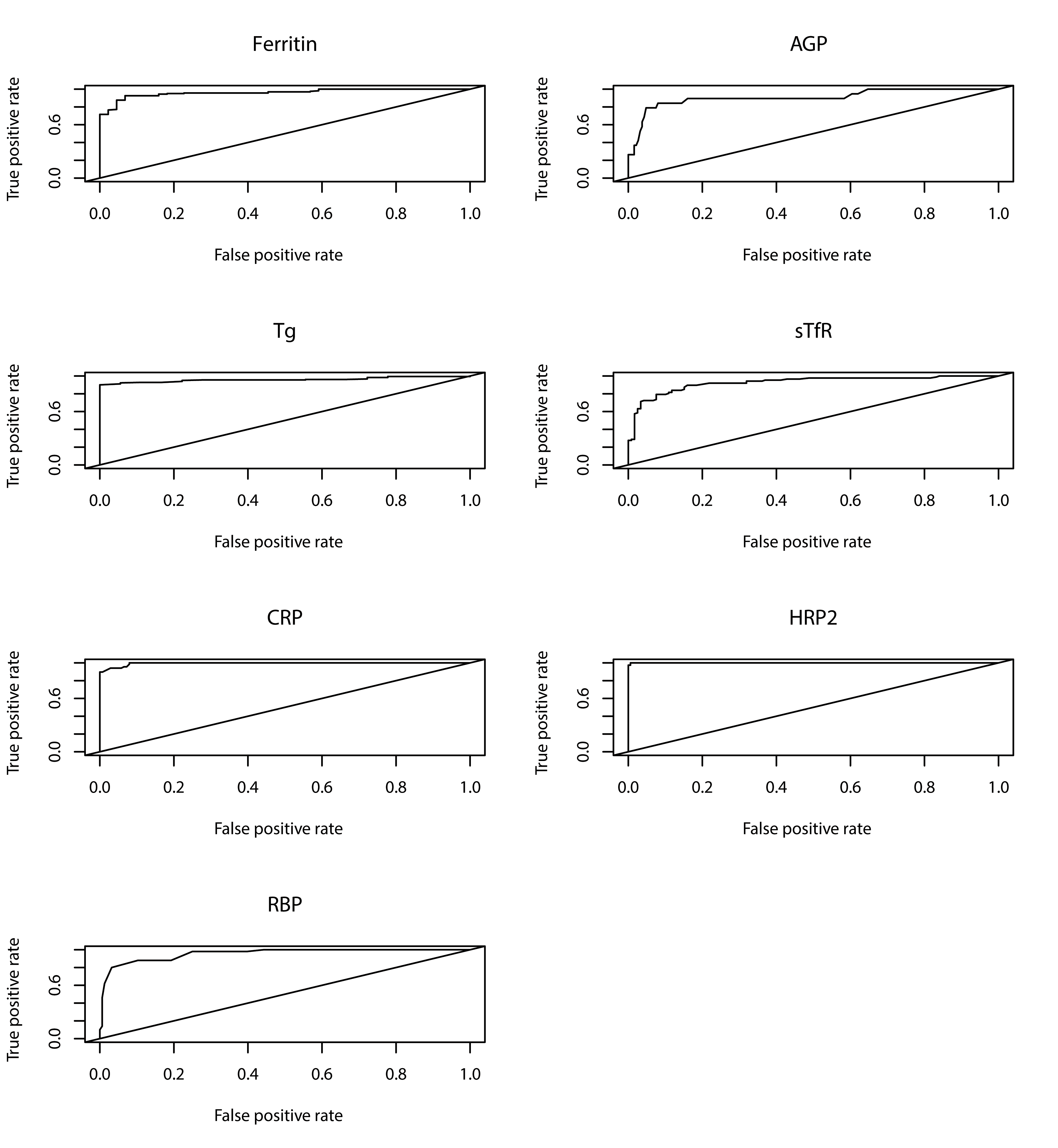

Supplement: S3 Fig — Receiver operating characteristic (ROC) curves plotting sensitivity (y-axis) against 1-specificity (x-axis) were generated from 7-Plex results of pregnant women participating in NiMaNu study to identify optimal threshold values for each analyte as measured in the 7-Plex to be used in specificity and sensitivity analysis. Optimal threshold values, defined as those resulting in maximal area under the ROC curves (AUC), are reported in Table 3. (TIF) [file pone.0185868.s003.tif]
