# Supplementary material for: Simultaneous assessment of iodine, iron, vitamin A, malarial antigenemia, and inflammation status biomarkers via a multiplex immunoassay method on a population of pregnant women from Niger
Source: PLoS One. 2017 Oct 5;12(10):e0185868. doi: 10.1371/journal.pone.0185868 (PMC5628875; doi:10.1371/journal.pone.0185868)
Supplement: S1 Table — (DOCX) [file pone.0185868.s004.docx]

**S1 Table.** **Summary statistics from the 7-Plex and conventional assays from USA donor training Panel and NiMaNu panel.**

| Analyte |  | USA Donor Panel | | NiMaNu Panel | |
| --- | --- | --- | --- | --- | --- |
|  |  | 7-Plex | Conv. | 7-Plex | Conv. |
| AGP (g/L) | Median | 0.67 | 0.53 | 0.42 | 0.42 |
|  | IQR | 0.18 | 0.18 | 0.27 | 0.38 |
|  | min - max | 0.16 - 1.2 | 0.32 - 1.04 | 0.14 - 1.1 | 0.1 - 2.66 |
|  | n | 72 | 72 | 206 | 206 |
| CRP (mg/L) | Median | 1.50 | 1.54 | 1.75 | 2.69 |
|  | IQR | 2.32 | 2.34 | 5.11 | 7.03 |
|  | min - max | 0.26 - 20.4 | 0.01 - 9.31 | 0.04 - 65.6 | 0 - 86.37 |
|  | n | 70 | 72 | 206 | 206 |
| Ferritin (µg/L) | Median | 33.80 | 30.38 | 34.80 | 34.90 |
|  | IQR | 59.50 | 40.97 | 71.90 | 57.83 |
|  | min - max | 0.82 - 694.1 | 7.52 - 375.89 | 0.53 - 1540.4 | 4.84 - 210.4 |
|  | n | 69 | 69 | 205 | 206 |
| HRP2 | Positive ^a^ | 0 | Not tested | 102 | 40 |
|  | Negative | 72 |  | 104 | 166 |
|  | n | 72 |  | 206 | 206 |
| RBP (µmol/L) | Median | 1.60 | 1.48 | 0.88 | 1.04 |
|  | IQR | 0.53 | 0.46 | 0.46 | 0.48 |
|  | min - max | 0.49 - 3.2 | 0.87 - 2.68 | 0.24 - 3.1 | 0.36 - 2.68 |
|  | n | 72 | 72 | 206 | 206 |
| sTfR (mg/L) | Median | 9.15 | 2.55 | 11.15 | 7.75 |
|  | IQR | 5.03 | 1.43 | 8.98 | 5.92 |
|  | min - max | 3.9 - 35.9 | 1.11 - 5.8 | 3.9 - 90.7 | 3.22 - 45 |
|  | n | 72 | 72 | 206 | 206 |
| Tg (µg/L) | Median | 18.00 | 15.07 | 14.90 | 32.41 |
|  | IQR | 20.70 | 18.62 | 15.93 | 23.33 |
|  | min - max | 0.89 - 207.2 | 2.1 - 231.36 | 2.2 - 110 | 7.14 - 148.7 |
|  | n | 70 | 68 | 206 | 190 ^b^ |

^a^ For the 7-Plex HRP2 assay, results are considered positive for HRP2 if values were above the assay limit of detection of 0.00212 µg/L. NiMaNu study HRP2 results were defined as positive for optical densities greater than 0.1415, ^b^ For Tg, 9 samples with extraordinarily high values (192.6-815.8 µg/L) in the NiMaNu study data were excluded. When those samples were included (n=199), the NiMaNu study Tg median was 32.41 µg/L (IQR 23.33 µg/L). IQR, interquartile range.
