# Supplementary material for: Simultaneous assessment of iodine, iron, vitamin A, malarial antigenemia, and inflammation status biomarkers via a multiplex immunoassay method on a population of pregnant women from Niger
Source: PLoS One. 2017 Oct 5;12(10):e0185868. doi: 10.1371/journal.pone.0185868 (PMC5628875; doi:10.1371/journal.pone.0185868)
Supplement: S2 Table — Median concentrations and interquartile range (IQR) of sTfR as measured by three immunoassay methods per sample set and comparisons between assays in absolute value (% recovery) and by Spearman correlation. (DOCX) [file pone.0185868.s005.docx]

**S2 Table. Performance comparison of different sTfR assays on both panels from USA donors and NiMaNu.** Median concentrations and interquartile range (IQR) of sTfR as measured by three immunoassay methods per sample set and comparisons between assays in absolute value (% recovery) and by Spearman correlation.

|  | sTfR assay | Median (IQR)mg/L sTfR | sTfR assay comparison | | |
| --- | --- | --- | --- | --- | --- |
|  |  |  |  | mean (SD) Recovery | Spearman correlation^a^ |
| USA training panel (n=72) | 7-Plex | 9.15 (5.03) | 7-Plex vs R&D Systems | 373% (108%) | 0.723 |
|  | R&D Systems | 2.55 (1.43) | 7-Plex vs Ramco | 173% (56%) | 0.610 |
|  | Ramco | 5.27 (3.19) | R&D Systems vs Ramco | 47% (10%) | 0.830 |
| NiMaNu panel (n=206) | 7-Plex | 11.15 (8.98) | 7-Plex vs R&D Systems | 533% (227%) | 0.841 |
|  | R&D Systems | 2.35 (1.32) | 7-Plex vs VitMin | 150% (49%) | 0.840 |
|  | NiMaNu  (VitMin Lab) | 7.75 (5.92) | VitMin vs R&D Systems | 353% (61%) | 0.969 |

^a^ All Spearman correlations were significant at the 0.01 level (2-tailed).
